# Alterations of membrane protein expression in red blood cells of Alzheimer's disease patients

**DOI:** 10.1016/j.dadm.2015.06.007

**Published:** 2015-07-21

**Authors:** György Várady, Edit Szabó, Ágnes Fehér, Adrienn Németh, Boglárka Zámbó, Magdolna Pákáski, Zoltán Janka, Balázs Sarkadi

**Affiliations:** a Institute of Enzymology, Research Centre for Natural Sciences, Hungarian Academy of Sciences, Budapest, Hungary; b Department of Psychiatry, Faculty of Medicine, University of Szeged, Szeged, Hungary; c MTA-SE Molecular Biophysics Research Group, Semmelweis University, Department of Biophysics and Radiation Biology, Budapest, Hungary

**Keywords:** Red cell membrane proteins, Biomarkers, Insulin receptor, GLUT1 transporter, ABCA1, ABCB6, ABCG2, PMCA, Erythrocyte membrane

## Abstract

Preventive measures, prognosis, or selected therapy in multifactorial maladies, including Alzheimer's disease (AD), require the application of a wide range of diagnostic assays. There is a large unmet need for relatively simple, blood-based biomarkers in this regard. We have recently developed a rapid and reliable flow cytometry and antibody-based method for the quantitative measurement of various red blood cell (RBC) membrane proteins from a drop of blood. Here, we document that the RBC expression of certain membrane proteins, especially that of the GLUT1 transporter and the insulin receptor (INSR), is significantly higher in AD patients than in age-matched healthy subjects. The observed differences may reflect long-term metabolic alterations relevant in the development of AD. These findings may pave the way for a diagnostic application of RBC membrane proteins as relatively stable and easily accessible personalized biomarkers in AD.

## 1 Introduction

Alzheimer's disease (AD) is manifested in a progressive dementia that currently affects over 40 million people worldwide. There are no reliable cures or disease-modifying therapies available, and there is no early diagnostic method to indicate AD before it has progressed to major memory loss and functional decline [1], [2]. Current biomarkers for this disease, including cerebrospinal fluid tau and amyloid-β determinations, MRI, and other imaging methods, are invasive, time-consuming, or expensive. New biomarkers for predicting the appearance, determining the progress, and reporting the effects of disease-modifying or preventative treatments are definitely required. Blood-based biomarkers may be an attractive option, but those available from among plasma proteins or lipids still need higher sensitivity and specificity. Measuring membrane proteins in blood cell samples may open a new avenue in this regard [3].

According to our recent work, the quantitative determination of human red blood cell (RBC) membrane proteins as biomarkers may offer new diagnostic possibilities. The expression and function of RBC membrane proteins for long has been suggested to provide information regarding various disease conditions and were considered as potential biomarkers [4]. As examples, in complex metabolic conditions, the RBC adrenergic receptor activation and a related adenosine triphosphate release, the Na-Li countertransport, or the alterations of glucose transport and the insulin receptor have been suggested to correlate with disease susceptibility, treatment response, or complications [5], [6].

We have developed a simple, rapid, and reliable flow cytometry-based diagnostic assay for the quantitative determination of the ABCG2 membrane protein in the human erythrocyte membrane [7]. In that work, we have documented a direct correlation between ABCG2 genotypes and RBC membrane expression levels, which may be useful to characterize patients potentially developing hyperuricemia and becoming afflicted with gout [7]. In a further study, we found low level RBC expression of the ABCB6 protein and documented a hereditary genetic basis for these altered expression levels [8]. Recently, we have further developed the panel of quantitative membrane protein detection in the RBC membrane, by examining several transporters and receptors through antibody binding in flow cytometry [9], [10]. These membrane proteins include the multidrug transporters ABCC1 and ABCC4, the glucose exchanger GLUT3, the sodium-glucose transporter SLC5A2 (SGLT2), the uric acid transporters SLC2A9 and URAT1 (SLC22A12), and PLSCR1 (a phospholipase scramblase). In as yet unpublished set of experiments, we performed quantitative measurements for these membrane proteins in numerous healthy subjects and in various multifactorial disease conditions with a strong metabolic background. An emerging picture based on these studies suggests a significant regulation of membrane protein expression under altered metabolic conditions, including type 2 diabetes and hyperuricemia/gout (unpublished data). To compile the potential RBC membrane biomarkers, we also performed mass spectrometry studies and generated a comprehensive, searchable database for the RBC membrane proteins [11].

In the present study, we have quantitatively analyzed the changes in the expression levels of several RBC membrane proteins in the groups of late and early onset AD patients, already characterized by a wide range of clinical and laboratory examinations. In this study, we have selected RBC membrane proteins based on a bioinformatics and network analysis of genome-wide association (GWA) studies, Online Mendelian Inheritance in Man (OMIM), and red blood cell (RBC) databases [11], [12] for a potential involvement in Alzheimer's disease. Here, we document that a limited number of transporters and receptors, potentially relevant in AD, show significant differences in RBC membrane expression in the AD patients as compared with normal, age-matched controls.

## 2 Methods

### 2.1 Selection of patients and controls

Forty patients diagnosed with Alzheimer's disease were included in this study. Twenty-seven of these patients were considered to have late onset AD (ages >70) and 13 patients were classified into early onset AD (age <61). All patients have been characterized by detailed clinical examinations, including imaging studies. For patient recruitment, clinical evaluation, and ethical permission, see the Supplementary Materials.

General laboratory diagnostic data were obtained at the University of Szeged, Hungary. For all AD patients and healthy individuals, the laboratory diagnostics were performed. The list of laboratory data obtained in this study and some of the key results are summarized in Supplementary Table 1.

RBC membrane protein determinations were performed by flow cytometry, according to our recently developed method [7], [8], [9], [10]. After antibody titration and using saturating amounts of selected antibodies, we quantitated the following red cell membrane protein levels: GLUT1 (SLC2A1), ABCA1, ABCB6, ABCG2, INSR, and PMCA4b. For the details of the RBC labeling, antibody sources, expression analysis, and statistics, see the Supplementary Materials.

## 3 Results

In all AD patients and control participants, detailed laboratory blood analytic examinations were performed. As documented in the Supplementary Table 1, the results of the laboratory tests showed no specific alterations between the patients and the control subjects. The only significant differences we found were somewhat increased granulocyte and decreased lymphocyte percentages and elevated alkaline phosphatase levels in the AD patients, probably reflecting the mild inflammatory-like conditions characteristic for this disease [1], [4]. Although some studies found altered levels in the major plasma lipids and lipid-binding proteins in AD patients [1], we did not observe such differences here.

**Fig. 1:**
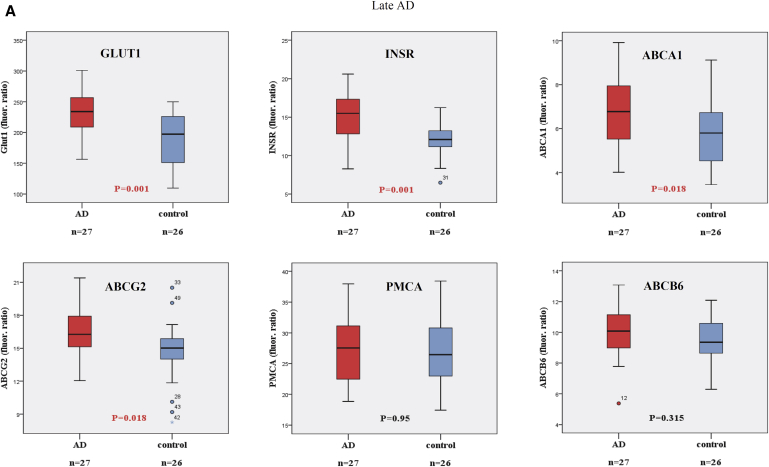

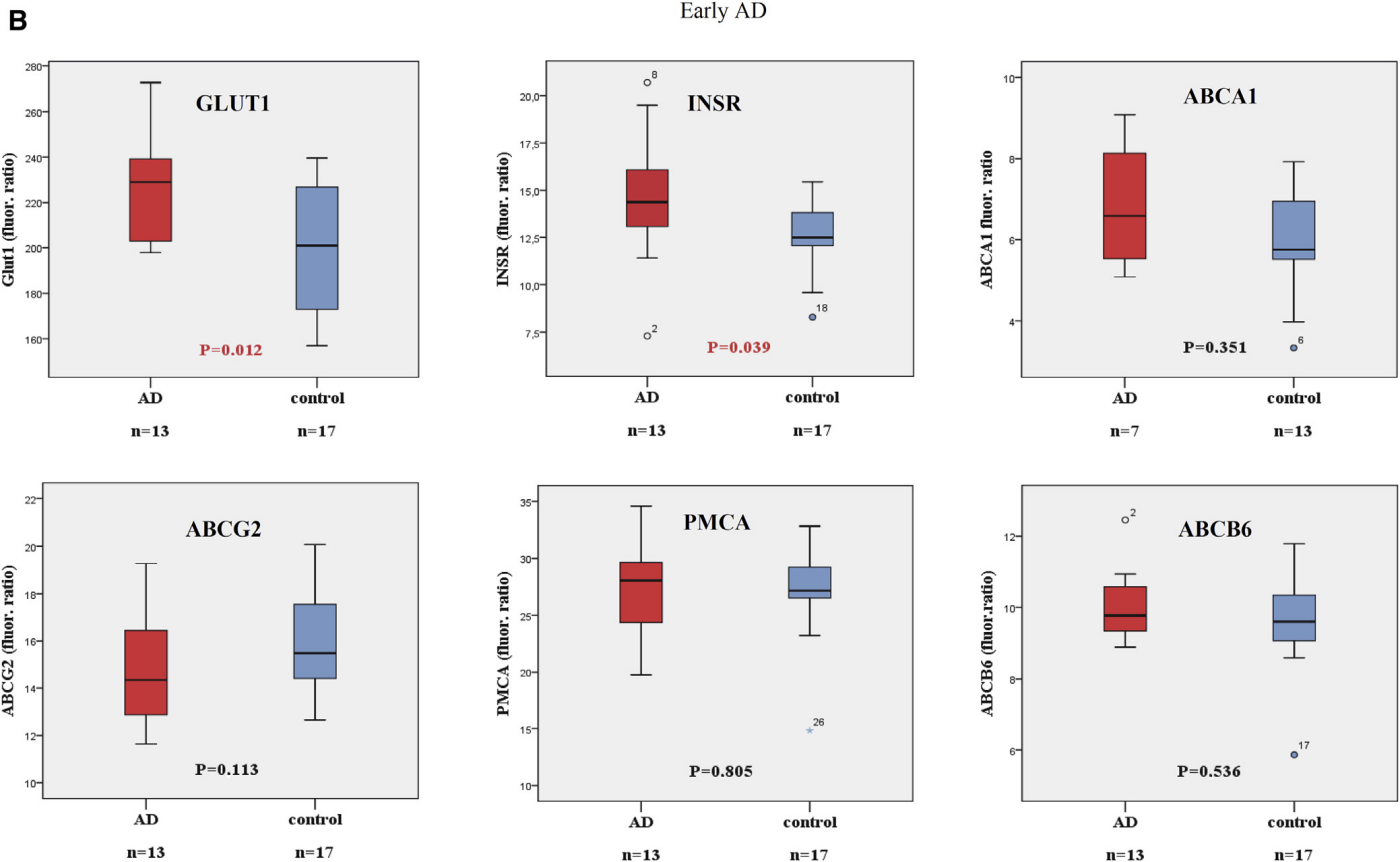
Expression of RBC membrane proteins in AD patients and age-matched control individuals, measured by flow cytometry. For details, see text and Supplementary Materials. Panel (A): Values obtained in late onset AD patients (ages >70 years) and age-matched controls. Panel (B): Values obtained in early onset AD patients (ages <61 years) and age-matched controls. (A) Late onset AD. (B) Early onset AD. Abbreviations: RBC, red blood cell; AD, Alzheimer's disease; PMCA, plasma membrane calcium pump.

In addition to the general clinical and laboratory examinations, we have examined the expression levels of several transporters and the insulin receptor in the RBC membranes of normal healthy and AD patients. The obtained data indicate that in late onset AD patients (Fig. 1A), the RBC membrane expression levels of the GLUT1 transporter and the insulin receptor (INSR) were significantly increased. In addition, we found significantly increased RBC membrane expression levels for the ABCA1 and the ABCG2 transporters, as compared with those in the age-matched control subjects. No measurable changes were observed in the expression levels of the plasma membrane calcium pump (PMCA) and the ABCB6 transporter.

In early onset AD patients (Fig. 1B), the results also showed a significant increase for the RBC membrane expression of GLUT1 and INSR, whereas we did not find significant alterations in the RBC membrane expression levels for ABCA1, ABCG2, PMCA, and ABCB6 between the early onset AD patients and the age-matched controls. When examining potential gender-related differences in these RBC membrane protein expression levels, we did not observe any significant differences either in the group of late or early onset AD patients and normal subjects (not shown).

## 4 Discussion

The mentioned data indicate major changes in the expression levels of metabolically relevant membrane transporters and a receptor in the RBC membrane of AD patients. The GLUT1 (SLC2A1) transporter is an abundant membrane protein in the human RBC, with an expression of about 100,000 copies per cell. The other main physiological sites of human GLUT1 expression are the blood-brain barrier endothelial cells, cardiac muscle, adipocytes, kidney cortex mesangial cells, and brain astrocytes. GLUT1 expression is tissue-dependently regulated by circulating glucose levels, hypoxia, as well as hormones, including insulin and growth hormones [13], [14], and it has been shown that chronic hyperglycemia increases RBC GLUT1 expression [15]. According to our present results, GLUT1 expression was significantly greater in both late and early onset AD patients than in age-matched controls, whereas systemic hyperglycemia was not apparent in the patients. Based on the relevant literature [1], [2], [16], we hypothesize that this increase in RBC reflects an upregulation of this transporter in the blood-brain barrier endothelial cells, caused by the relative metabolic starvation and/or hypoxia in the brain tissues.

The insulin receptor (INSR) in the human RBC was shown to have the same basic features as in other tissues [17], a reduced binding of insulin to RBC-INSR was observed in non–insulin-dependent diabetes, and in hypertension with hyperinsulinemia [18]. In the present study, we observed a significant increase in RBC-INSR in both late and early onset AD patients. This may reflect an overall upregulation of the INSR expression, caused by the relative insulin deficiency in the central nervous system (CNS) [1], [2].

The ABCA1 protein is involved in the formation of high-density lipoprotein (HDL) cholesterol and the reverse cholesterol transport from the liver and various cell types [19] and is present in the human RBC [9], [10], [11]. Here, we found that in late onset AD, this protein is significantly increased in the RBC membrane, which may reflect a long-term regulatory response in cholesterol transport, not apparent in the actual plasma cholesterol or binding protein levels.

The ABCG2 multidrug transporter has a role in xenobiotic transport and cancer drug resistance, uric acid transport, and was identified as the junior blood group component [20]. We have shown a cosegregation of low RBC-ABCG2 expression with mutant variants [7], whereas here, we found that in the late AD, ABCG2 expression was significantly greater than in the age-matched controls. An earlier study from our group found lower prevalence of the Q141K-ABCG variant in AD patients [21], whereas a higher protein expression in RBC, shown here, may reflect a general upregulation of ABCG2, indicated to play a role in protecting the CNS against amyloid accumulation [22]. In this study, we found that the RBC membrane expression of PMCA4 (the “housekeeping” calcium pump) and ABCB6 (the Lan blood group component) was unchanged in AD.

The observed increase in the GLUT1, INSR, ABCA1, and ABCG2 expression in the RBC membrane of late onset AD patients may indicate underlying metabolic alterations and probably a general upregulation of these proteins also in tissues related to the CNS, reflecting systemic transcriptional or translational effects. The increase in GLUT1 and INSR in early onset AD suggests similar metabolic alterations in this disease. Obviously, further and larger cohort studies are required to establish a relationship of the expression of these RBC membrane proteins with treatment protocols, therapeutic responses, or complications in AD. Because the membrane proteins studied here have also been implicated in major metabolic diseases, including type 2 diabetes and gout, overlapping disease mechanisms may be also reflected by the RBC membrane biomarkers. In our preliminary, as yet unpublished studies, we found that in insulin-resistant type 2 diabetes patients, the expression of GLUT1 in RBC is significantly upregulated, whereas the level of INSR, in contrast to that in the AD patients, is greatly reduced. Of course, further detailed studies are required to appreciate these changes, and the determination of additional RBC membrane proteins may provide a panel for differential diagnostics of various metabolic alterations.

Interestingly, the red cell membrane contains more than 300 different membrane proteins, ([11] http://rbcc.hegelab.org/) among which several may be diagnostically relevant in AD. These include the amyloid-β-A4protein, α-synuclein, presenilin-1, nicastrin, acetylcholinesterase, ABCA7, phospholipid-scramblases, or the GLUT3 transporter. We are currently installing quantitative assays for these further RBC membrane proteins, whereas the limited availability of specific, high affinity antibodies and the costs for purchasing and selecting such antibodies are hindering these efforts. Still, based on the easy accessibility of red cells, this methodology may provide new possibilities for membrane protein biomarker diagnostics in AD and related multifactorial diseases.

**Research in context**

1. Systematic review: To follow progression and potential therapeutic effects, the laboratory diagnostics of Alzheimer's disease (AD) requires multiple, preferably noninvasive, and inexpensive new tools. Blood-based biomarkers may serve this need.
2. Interpretation: Our current data indicate that a quantitative determination of the expression level of certain red blood cell (RBC) membrane proteins by flow cytometry may offer new potential biomarkers in AD. We document that the expression of the GLUT1 sugar transporter and the insulin receptor in the RBC membrane of the AD patients is significantly increased. Similar tendency is observed for the ABCA1 lipid transporter and the ABCG2 multidrug transporter.
3. Future directions: A detailed analysis of the expression of these and several more RBC membrane proteins may help to construct a relatively simple and inexpensive diagnostic panel for supporting AD diagnostics. This technology may also help to understand the pathologic regulatory effects underlying this disease.
