# Supplementary material for: Alterations of membrane protein expression in red blood cells of Alzheimer's disease patients
Source: Alzheimers Dement (Amst). 2015 Jul 21;1(3):334–8. doi: 10.1016/j.dadm.2015.06.007 (PMC4878320; doi:10.1016/j.dadm.2015.06.007)
Supplement: Supplementary Materials [file mmc1.docx]

**Supplementary Materials**

**for Várady et al “Alterations of membrane protein expression in human red blood cells in Alzheimer’s disease patients”**

1. AD patients

AD patients were recruited from the Memory Clinic of the Department of Psychiatry, University of Szeged, Hungary. Initial examination of AD patients included the evaluation of personal and family medical history, neurological and psychiatric examination with the assessment of psychometric tests to confirm cognitive impairment. The clinical diagnosis of probable AD was established according to the criteria of the National Institute of Neurological and Communicative Disorders and Stroke/Alzheimer’s Disease and Related Disorders Association (NINCDS/ADRDA) [[1](#_ENREF_1)]. The cognitive evaluation of AD patients was carried out using the AD Assessment Scale-Cognitive Subscale, the Mini-Mental State Exam (MMSE) and the Clock Drawing Test.

The study was approved by the Committee of Science and Research Ethics (ETT TUKEB reference 53062/2012/EKU, - 801/PI/12) and all patients or their guardians provided written informed consent.

2. Laboratory data for the patients included were obtained for Na^+^, K^+^, Cl^-^, glucose, total serum protein, albumin, cholesterol, triglyceride, HDL, LDL, GOT, GPT, ALP, GGT, eGFR, carbamide, creatine, uric acid, iron, WBC, neutrophil %, lymphocyte %, monocyte %, eosinophil %, basophil %, RBC, HGB, HTC, MCV, MCH, MCHC, RDW, platelet count, MPV, TSH, freeT3 and CRP.

**Supplementary Table 1.** Summary of some key laboratory data for AD patients and age-matched control subjects

|  | glucose (mmol/l) | WBC (Giga/L) | Neutrophyl % | Lymphocyte % | Monocyte % | Eosinophil % | Basophil  % | RBC (T/L) |
| --- | --- | --- | --- | --- | --- | --- | --- | --- |
| Late AD | 5.6 | 6.8 | 63.5 | 25.2 | 7.9 | 2.9 | 0.6 | 4.49 |
| Late Control | 5.5 | 6.8 | 58.4 | 30.3 | 7.6 | 3.2 | 0.4 | 4.62 |
| SignificanceP value | NS  p=0.671 | NS  p=0.912 | **S**  **p=0.025** | **S**  **p=0.022** | NS  p=0.627 | NS  p=0.7 | NS  p=0.336 | NS  p=0.203 |
| Early AD | 6.5 | 7.2 | 61.7 | 28.5 | 7.8 | 1.3 | 0.6 | 4.76 |
| Early Control | 6.2 | 7.1 | 61.9 | 27.9 | 6.9 | 2.8 | 0.6 | 4.85 |
| SignificanceP value | NS  p=1.000 | NS  p=0.68 | NS  p=0.805 | NS  p=0.621 | NS  p=0.245 | **S**  **p=0.004** | NS  p=0.711 | NS  p=0.457 |
| Laboratory reference range | 3.3-5.6 | 3.9-11.1 | 44-68 | 25-29 | 5.0-6.0 | 1.5-7.5 | 0.0-3.0 | 3.88-4.99 |

3. Flow cytometry analysis

Whole blood samples were obtained in Vacuette tubes containing EDTA (Greiner Bio-One) at the hospital. 50 µl human blood was diluted in 4ml phosphate buffered saline (PBS) containing 1% paraformaldehyde (PFA) and fixed at 37˚C for 5 minutes. The cells were centrifuged at 1,000×g for 5 min and the pellet was resuspended in 150 µl PBS. Alexa Fluor-647 conjugated wheat-germ agglutinin binding (by using 1 µg/mL WGA-Alexa Fluor-647), and antibody staining were performed for 40 min at 37°C by using the following mouse anti-human monoclonal antibodies: anti-ABCG2 (Clone: Bxp34 final concentration 5 µg/ml), anti-glucose transporter-1 (Glut-1, final concentration 2.5 µg/ml, R&D Systems), anti-insulin receptor mouse monoclonal (INSR, final concentration 50 µg/ml, Thermo Scientific Pierce), anti-ATP binding cassette transporter A1 mouse monoclonal (ABCA1, final concentration 40 µg/ml, Abnova), anti-plasma membrane calcium pump 4b (PMCA, Clone: JA3, final concentration 4 µg/ml), anti-ABCB6 monoclonal humanized antibody (see ref. [[2](#_ENREF_2)] - final concentration 5 µg/ml) and isotype control antibodies (IgG2b, IgG1, final concentration 5 µg/ml, Life Technologies).

After washing out primary antibodies, secondary antibodies corresponding to the IgG type and labeled with phycoerythrin (PE) were added to the cells (Goat F(ab’)2 fragment anti-mouse IgG-PE (GAM IgG2b-PE, GAM IgG1-PE, final concentration 20 µg/ml, Life Technologies), incubated for 30 min at 37^o^C, washed and resuspended in PBS. Phycoerythrin conjugated goat anti-human secondary antibody (Goat F(ab’)2 fragment anti-human IgG-PE, final concentration 40 µg/ml, Beckman Coulter) was used in case of ABCB6. The labeled samples were subjected to flow cytometry (FACS); intact red cells were gated based on the forward scatter (FSC) and side scatter (SSC) parameters. In addition, RBC ghosts were gated by WGA Alexa Fluor-647 staining, thereby separated from background. Intact cells were analyzed for antibody staining by a FACSCanto II flow cytometer (excitation wavelengths: 488 nm (solid state laser) and 633 nm (HeNe laser), emission filters: 582/42 nm for PE and 660/20 for Alexa Fluor-647).

The labeling of the RBC membrane proteins in each case provided a well defined peak, easily distinguished from the isotype controls (see Supplementary Figure 1). Saturation of the labeling was assured by antibody titrations, specificity was analyzed by the use of specific monoclonal antibodies from various sources and generated against various epitopes (see [[3](#_ENREF_3)], [[4](#_ENREF_4)]). In each individual two parallels were measured; relative antibody expression was calculated by dividing the median values obtained with the primary and secondary antibodies with the weighted average of the median values obtained with isotype control and secondary antibodies. The weighted average (WA) was calculated as follows: WA = (average isotype control fluorescence of all measured samples*2+individually measured isotype control fluorescence)/3.

The calculated values of individuals were statistically analyzed by Mann-Whitney U-test (IBM SPSS ver.21).

**Supplementary Figure 1.** Flow cytometry analysis of the expression of the GLUT1 and the INSR proteins in human red blood cells.

Panel A. Panel B.


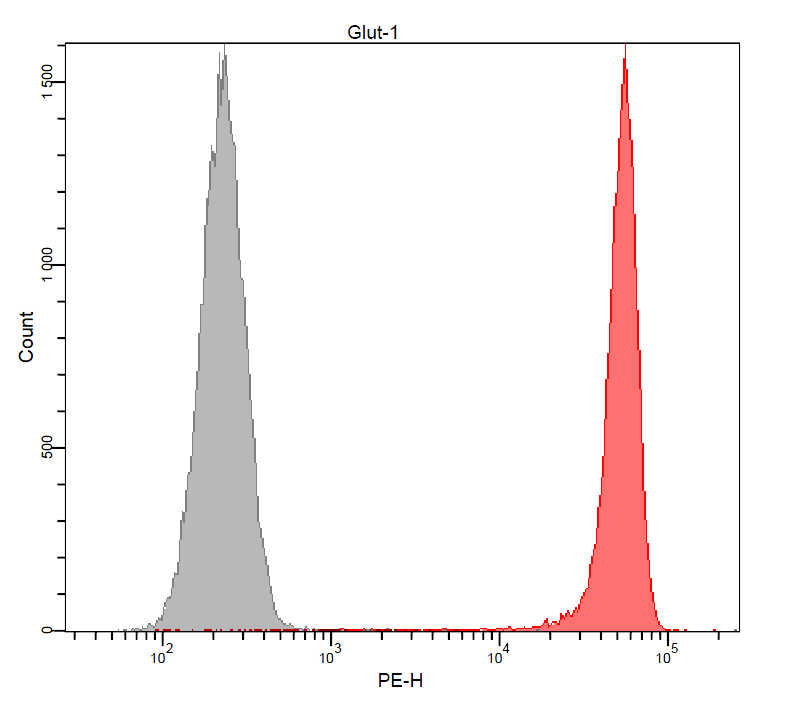

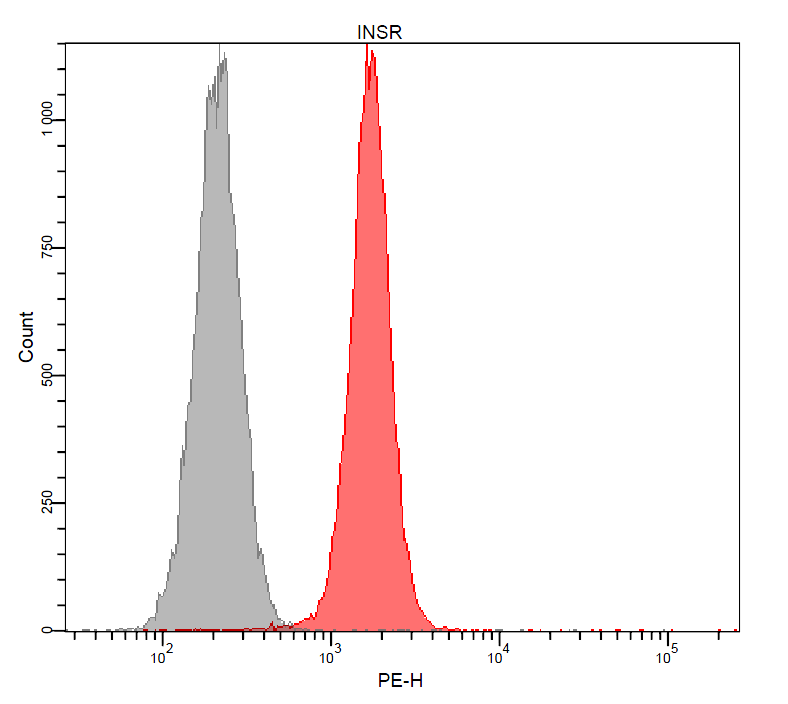


**Panel A.** Expression of the GLUT1 protein in human red blood cells. Detection of the GLUT1 protein by MAB1418 (R&D Systems) monoclonal antibody

**Panel B.** Expression of the insulin receptor (INSR) protein in human red blood cells. Detection of the insulin receptor by insulin receptor (beta subunit) monoclonal antibody [18-44] (Ab983, Abcam).

Flow cytometry was carried out in fixed and permeabilized human erythrocytes (see Methods). Grey histograms: IgG2b isotype control, red histograms: specific mAb.

**References**

1. McKhann, G., et al., *Clinical diagnosis of Alzheimer's disease: report of the NINCDS-ADRDA Work Group under the auspices of Department of Health and Human Services Task Force on Alzheimer's Disease.* Neurology, 1984. **34**(7): p. 939-44.

2. Koszarska, M., et al., *Screening the expression of ABCB6 in erythrocytes reveals an unexpectedly high frequency of Lan mutations in healthy individuals.* PLoS One, 2014. **9**(10): p. e111590.

3. Kasza, I., et al., *Expression levels of the ABCG2 multidrug transporter in human erythrocytes correspond to pharmacologically relevant genetic variations.* PLoS One, 2012. **7**(11): p. e48423.

4. Varady, G., et al., *Cell surface membrane proteins as personalized biomarkers: where we stand and where we are headed.* Biomark Med, 2013. **7**(5): p. 803-19.
